# A novel approach for mapping exposure to land cover at the small statistical geography level

**DOI:** 10.1186/s12942-025-00425-7

**Published:** 2025-11-25

**Authors:** Joanne K. Garrett, Lewis R. Elliott, Rebecca Lovell, Benedict W. Wheeler, Tom Marshall, Fränze Kibowski, Benjamin B. Phillips, Kevin J. Gaston

**Affiliations:** 1 European Centre for Environment and Human Health, University of Exeter Medical School, Penryn, UK; 2 Natural England, Foss House, York, UK; 3 Environment and Sustainability Institute, University of Exeter, Penryn, UK

## Abstract

Many studies linking spatial environmental exposures to health outcomes rely on small statistical geography units, such as Lower-layer Super Output Areas (LSOAs), to estimate exposure. However, these units commonly vary in size, particularly between urban and rural areas, leading to potential exposure misclassification. This study proposes a new method for better capturing environmental exposure at the small statistical geography unit level. Using the Living England Habitat Map as an example, we combined LSOA and postcode-level data to account for varying area sizes and mitigate edge effects. We compared our method with the typical approach, which calculates an average at the small geography unit level. Overall, our proposed method resulted in lower exposure to non-built-up areas compared to averaging across entire LSOAs, whereas exposure to built-up areas was higher by 8–10%. However, these patterns varied based on region, urban/rural classification, land cover type, and LSOA size class. We suggest that this proposed method offers a more consistent approach to estimating neighbourhood exposure to nature.

**Supplementary Information:**

The online version contains supplementary material available at 10.1186/s12942-025-00425-7.

## Introduction

There is a growing body of research investigating the links between green and blue spaces and human health and well-being [1, 2]. Much of the population level research has relied on linking environmental data with human health and well-being data at relatively coarse spatial levels. For example, data is often linked at the small statistical geography level [3, 4] in the UK [5–17] and similarly in Europe [18], Australia [19], North America [20, 21] and China [22, 23]. Within small area statistics, exposure metrics can be ascribed to individuals or populations based on quantity, for example the percentage coverage of green space within the area [19], or access, for example distance to the coast [5, 24].

Other methods can be used at finer spatial resolutions when, for example, home locations are known, such as metrics within a buffer of a home coordinate or postcode centroid, or proximity to home by network distance [25–29]. However, such precise geolocation is often unavailable or restricted to protect anonymity [30]. Researchers therefore make implicit or explicit assumptions that small statistical geography units or administrative boundaries sufficiently capture the environmental conditions that people are exposed to and experience in their everyday lives [31, 32]. There are a number of typical limitations based on these assumptions, especially around exposure misclassification, the ecological fallacy (drawing individual conclusions from group data), the modifiable areal unit problem, and not considering human mobility and the amount of time spent outside [33–36].

One key issue is that these statistical geography units typically vary substantially in size, given marked variation in population density, and that units are often designed to have roughly equivalent population counts. This can mean that within a single dataset, the potential for environmental exposure misclassification for the population of each small area can be highly variable, especially between urban and rural areas [33]. An urban resident living in a geographical unit with a very small area may frequently be exposed to environmental conditions beyond the boundaries of that unit. Conversely, a rural resident of a very large geographical unit may only typically experience the environmental conditions within a small fraction of the area covered by that unit.

As an illustration, in England, Lower Layer Super Output Areas (LSOAs) are often used in reporting statistics such as census and population health data. LSOAs are designed to have approximately similar population size (approximately 1,500 people per LSOA), leading to spatial size variability between rural (mean area ~ 19 km^2^) and urban (mean area ~ 1 km^2^) areas, with a very large overall range from 0.02 to 684 km^2^. For individuals in larger rural LSOAs, the overall characteristics of the LSOA of residence may not reflect their immediate environmental exposures. Conversely, individuals in smaller urban LSOAs may experience a larger area than their LSOA. Although the use of a population-weighted centroid attempts to account for residential distribution within the LSOA [24, 37], it may still inaccurately represent exposures for households, particularly in rural or larger LSOAs (i.e. it assumes every resident lives at the population weighted centroid). Such exposure misclassification could lead to incorrect conclusions about the relationship between exposures and outcomes. For example, Tian et al. [38], found an association between green-ness and mortality when considering large areas around the home and no significant association when considering smaller areas. Similarly, associations between green-ness and self-reported health in New York varied according to the resolution of the underlying vegetation data and the area unit used [39]. Nevertheless, linking individual-level data to environmental factors often requires aggregation at the small statistical geography level. In an improved approach, Mears et al. [9], averaged the number of trees within a 100 m buffer of each residence within an LSOA for a city in the UK and found that different greenspace indicators were significantly associated with distinct mental health and well-being outcomes.

A range of environmental products have been linked with individual or aggregate health or behaviour metrics at the small statistical geography level to gain an understanding of the variety of types and qualities of nature potentially available to people in their neighbourhood, such as the UKCEH Land Cover Map [12, 13], as well as other remote sensing derived land cover maps [15]. This study utilises a similar product (the Living England Habitat Map) to demonstrate a new method of characterising neighbourhood exposure to the types and qualities of nature. Our proposed method uses data at both LSOA and postcode (sub-LSOA) levels for England, first calculating the percentage coverage of land cover types within 300 m postcode buffers, then averaging these at the LSOA level weighted by the number of domestic postal delivery addresses (as a proxy for population per postcode). It mitigates edge effects by allowing habitat exposure to extend beyond the LSOA boundary through use of a 300-metre postcode buffer and maintains consistency across varying LSOA sizes. We argue that the new proposed approach reduces the potential for exposure misclassification associated with variable unit size at the small statistical geography level.

## Method

Throughout the paper we’ll refer to the two methods as the “proposed method” and the “typical method”. The proposed method consists of calculating the percentage coverage of land cover types within 300 m postcode buffers, then averaging these at the LSOA level weighted by the number of domestic postal delivery addresses. We compare this approach with a typical method, which involves calculating the percentage cover of land cover types within each LSOA.

All processing and analyses were carried out in RStudio (Build 764; 2024; Posit Software, PBC) with R version 4.3.3 (R Development Core Team 2024) and packages “sf” [40] and “terra” [41]. The code is available on Github at github.com/j-k-garrett/RENEW_mapping.

### Data

#### LSOAs and postcodes

LSOAs were obtained for the year 2011 [42] (Supplemental Fig. 1). Postcode locations were obtained from the UK’s Ordnance Survey dataset of postcode locations (Code-Point; [43]), which is accessible through the EDINA Digimap service for UK educational and research institutions. The Code-Point dataset is updated quarterly, such that any new housing developments are included [45]. Postcodes usually include multiple addresses (median = 12, mean = 17 dwellings or ‘domestic delivery points’), so the postcode location provided is the coordinates of the nearest delivery point (i.e. postal address) to the calculated mean position of the delivery points in the postcode [45]. The postcode location dataset provides the number of domestic delivery points (primarily individual dwellings) associated with each postcode, such that delivery points associated with organisations are excluded and domestic residences are included. Only English LSOAs and postcodes were retained for further processing due to correspondence with the environmental data used (see followingsection "Land cover"). LSOAs were assigned urban/rural status using the 2011 ONS rural/urban classification [46]. Census output areas (OAs; smaller units which are built into LSOAs) are assigned urban/rural classification based on whether the majority of their population lives in a settlement of 10,000 people or more, using physical settlement boundaries created by Ordnance Survey (OS). LSOAs are then assigned urban/rural classification based on the majority of their constituent OAs [47]. “Town and fringe” LSOAs are included within the “Rural” category. We assigned each LSOA to an English region using the Ordnance Survey dataset Boundary-Line [48] based on the location of its centroid.

#### Land cover

The Living England Habitat Map (Supplemental Fig. 2) is a probability-based map showing the extent and distribution of broad habitats across England [49]. It has been created using a range of satellite imagery products from 2021 to 2022, where the smallest spatial resolution is 10 m [49], combined with field data records and other geospatial data in a machine learning framework. An average habitat classification accuracy of 88% was reported (comparing the map-based probability with field collected data), although this accuracy varies with region and habitat. The Living England Habitat includes 17 habitats. These are: acid, calcareous or neutral grassland; arable and horticulture; bare ground; bare sand; bog; bracken; broadleaved, mixed and yew woodland; built-up areas and gardens; coastal saltmarsh; coastal sand dunes; coniferous woodland; dwarf shrub heath; fen, marsh and swamp; improved grassland; scrub; unclassified; and water.

The Living England Habitat Map does not include features such as estuaries. As such, estuaries and rias were obtained from the Coastal Physiographic features product from JNCC [50, 51]. These two datasets were merged and clipped to the boundary of the Living England Habitat map to ensure no overlap (Supplemental Fig. 3).

English postcodes located near the Scottish and Welsh borders can extend within 300 m into these neighbouring regions. To address this, boundary data for these areas were obtained from the Ordnance Survey dataset Boundary-Line [48] (Supplemental Fig. 3). This was also clipped with the Living England Habitat map to ensure no overlap. A total of 51 LSOAs (0.16%) had postcode buffers that extended into Scotland and Wales. Of these, the median percentage coverage of Scotland and Wales was 1% of the averaged LSOA area. This area was excluded when calculating the percentage coverage of land covers, such that only area in England was considered.

A shapefile for the sea was created by merging the Living England boundary, the estuaries and rias dataset and the Scotland and Wales dataset, adding a 500 m buffer and then finding the difference (Supplemental Fig. 3).

### Calculating land cover percentage coverage by LSOA

**Fig. 1:**
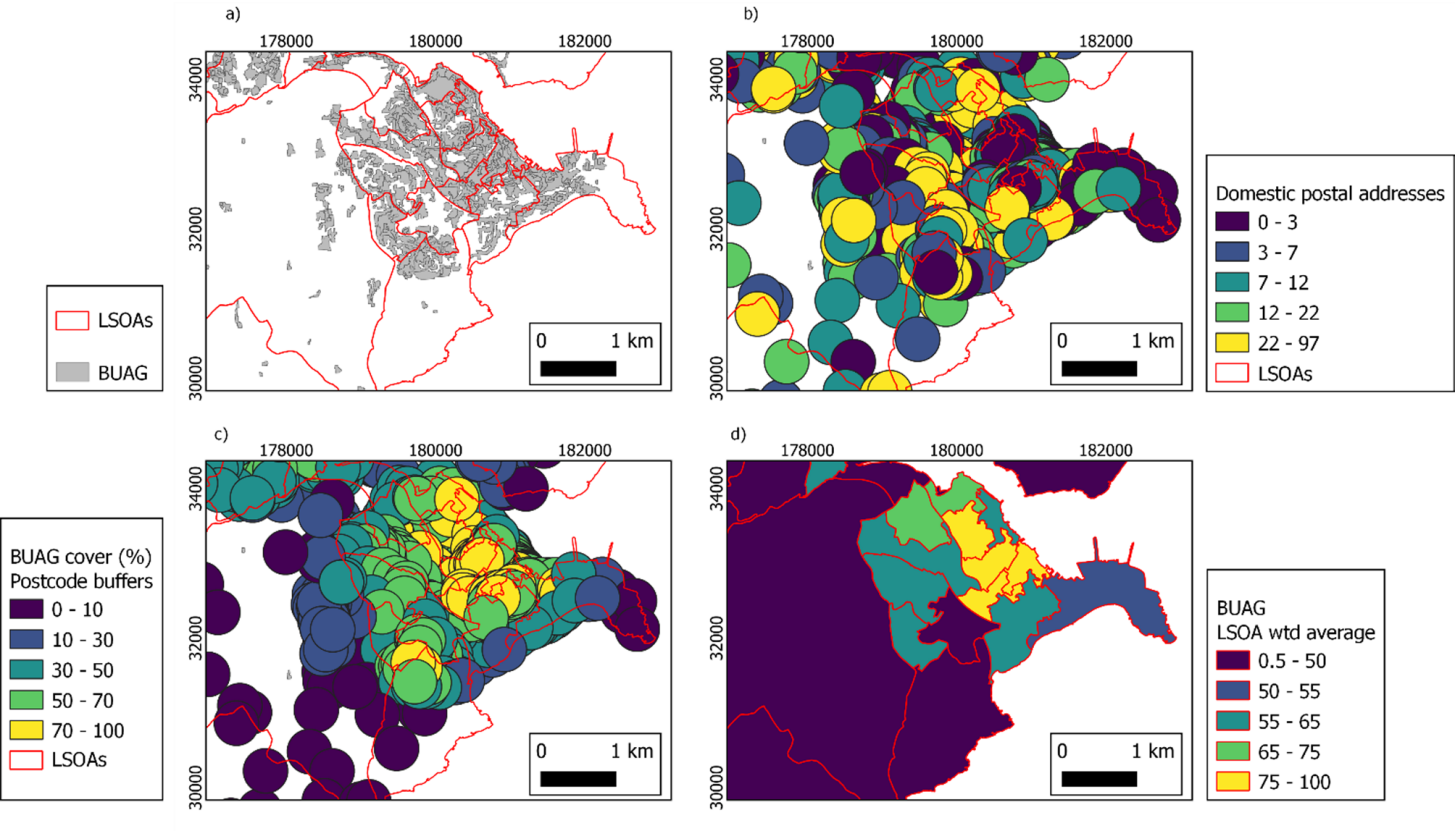
Falmouth area including urban and more rural Lower-layer super output areas (LSOAs) (**a**) Built-up area and gardens from the Living England habitat map [60]; (**b**) 300 m buffers around postcode point locations coloured by the number of domestic postal addresses [43]; (**c**) Built-up area and gardens percentage cover of each postcode buffer; d) LSOA average Built-up area and gardens, weighted by the number of domestic postal addresses. Coordinates are British National Grid

To calculate the proposed method, where the percentage coverage of different land covers within 300 m postcode buffers were averaged at the LSOA level and weighted by the number of domestic delivery addresses, postcode points were first joined with LSOA information. We used postcode point locations and the number of domestic delivery addresses as a proxy of population distribution to indicate where people actually live, as in Norman et al. [52], and Brindley et al. [53],. Then a 300 m buffer was calculated for each postcode point (“st_buffer”; package “sf”). A 300 m buffer was selected as this corresponds with policy measures indicating people should have accessible green spaces within 300 m of their home such as the WHO’s accessible urban greenspace indicator, Natural England’s Local Accessible Natural Greenspace target and the 3–30-300 rule [54–57]; it is considered to approximate to a 5 min walk and visit frequency has been found to decrease beyond this distance [58]. It has also been used in previous research [15, 27, 59]. The percentage coverage of each habitat type, estuaries and rias, and the sea within each 300 m buffer was then calculated for each postcode. Area within Scotland and Wales was excluded. For each LSOA, the mean of each percentage coverage was then calculated from every component postcode located within the LSOA and weighted according to the number of domestic delivery addresses within each postcode. Figure 1 demonstrates an illustrative example of LSOAs of different sizes; (a) the underlying Living England land cover data for an example land cover (built-up area and gardens); (b) The 300 m postcode buffers coloured by their number of domestic postal addresses; (c) the 300 m postcode buffers coloured by the percentage of built-up area and gardens within them; and (d) the resulting domestic postal address weighted LSOA percentage cover of built-up area and gardens.

Second, the more typical approach to allocating land cover to LSOAs was applied, calculating the percentage coverage of each habitat type (and estuaries/rias, and the sea) within each LSOA (“st_intersect”; package “sf”).

### Comparative analysis

To compare between the estimates of land cover as calculated by the two different methods, we used the Kolmogorov-Smirnov test to assess statistical differences in the distribution. This test makes no assumptions about the underlying statistical distributions of the data.

We tested the correlation between habitat coverage values obtained by the two different methods using Kendall’s tau. This statistical measure is chosen for its robustness when dealing with multiple occurrences of the same value, which in our case were often zeros. We visualised the correlation with a 2D density plot (function “geom_bin2d”) and visualised the relationship with a generalised additive model (GAM) smoothing function. GAMs also make no assumptions about the distribution function of the relationship between the two methods, enabling visualisation of non-linear relationships.

Finally, we present distributions within different size classes of LSOAs and applied the Mann-Whitney U test to assess whether one method tends to have higher or lower values than the other. The size class distribution is highly right-skewed, this means that there are more small LSOAs than larger ones. To represent this spread, we define size classes using a logarithmic scale. The smallest class contains the greatest number of items, we therefore further subdivide it to capture more detail.

## Results

### LSOAs

**Fig. 2:**
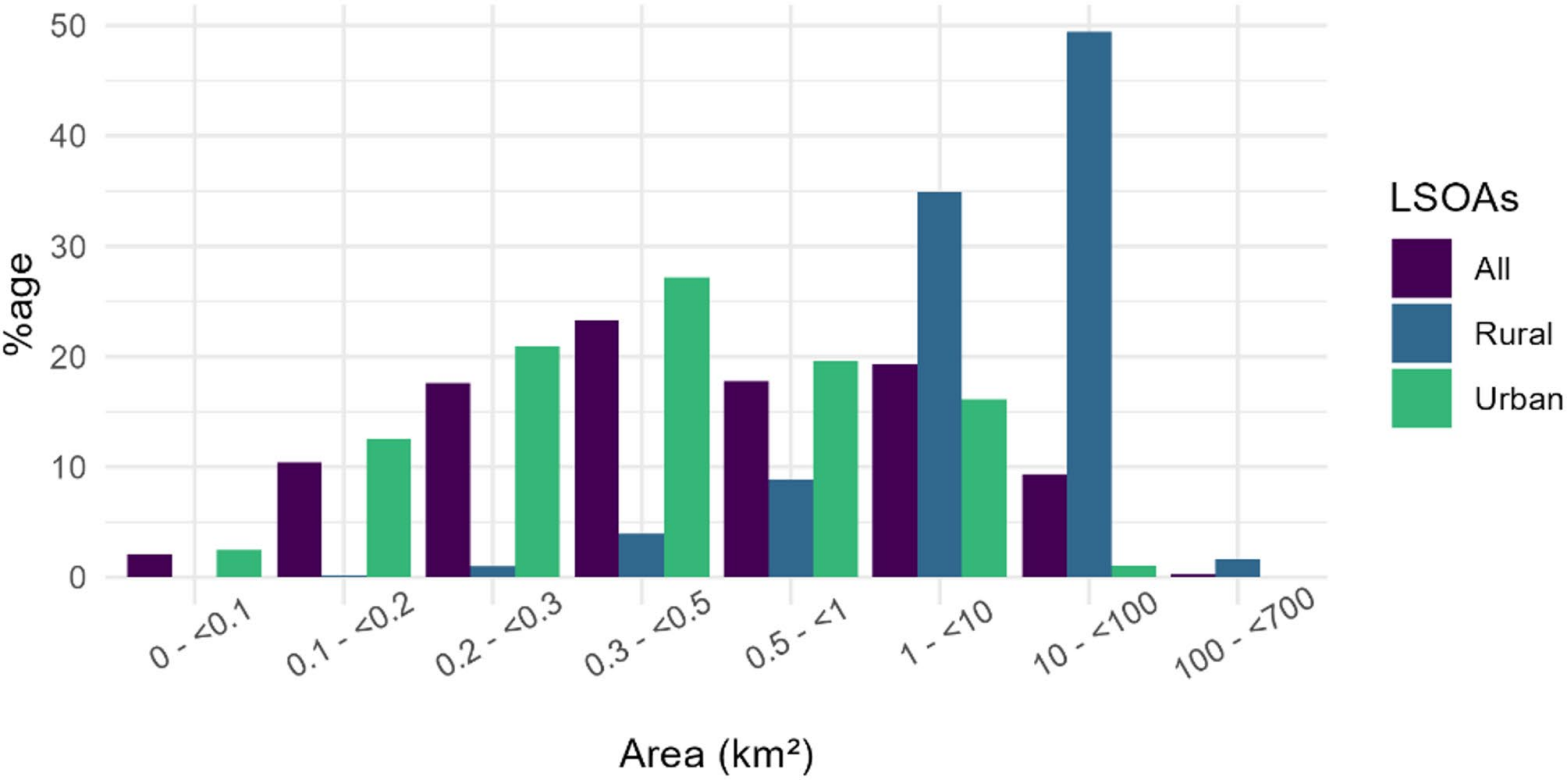
Distribution of LSOA area across England, all combined and rural and urban separately

There was a total of 32,884 LSOAs included in the calculations. LSOAs ranged in size from 0.018 km^2^ to 684 km^2^ and the median LSOA size was 0.46 km^2^. On average, rural LSOAs were considerably larger than urban LSOAs (Table 1; Fig. 2), although there were considerably more urban LSOAs (83%) as compared to rural LSOAs (17%), reflecting the urban/rural distribution of the population.

Figure 1 illustrates that, based on our proposed methodology, LSOAs are allocated land cover from beyond their own boundary (i.e. where postcode buffers extend beyond the LSOA boundary), and also that land cover in some areas of the LSOA does not contribute data at all (i.e. areas of the LSOA beyond 300 m from a postcode centroid). As intended, this means that land cover is allocated to the LSOA population based on where people actually live regardless of the LSOA’s boundaries, and where residences are close to the boundary they are allocated land cover exposure from beyond that boundary.

**Table 1:** Descriptive statistics for all LSOAs, and rural and urban LSOAs separately

LSOAs	*N*		LSOA area (km^2^)			
		Median	Mean	SD	Min	Max
Total	32,844	0.46	3.97	13.35	0.02	684.23
Rural	5,598	10.50	18.88	27.50	0.13	684.23
Urban	27,246	0.38	0.91	2.12	0.02	53.66

### Descriptive statistics and maps

**Fig. 3:**
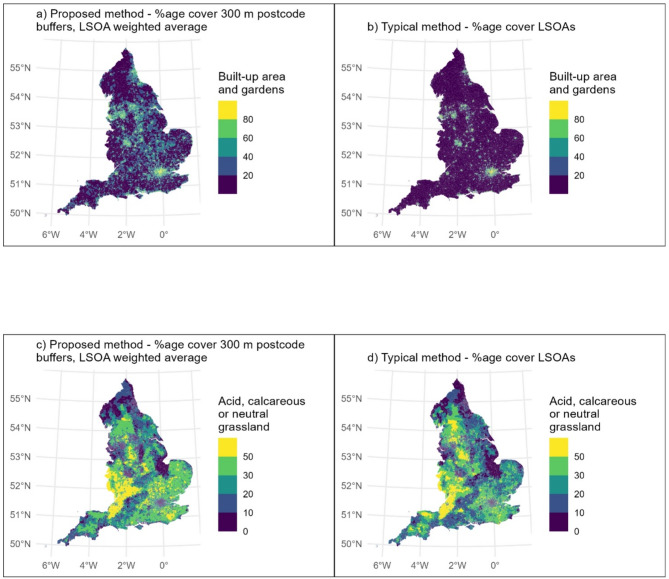

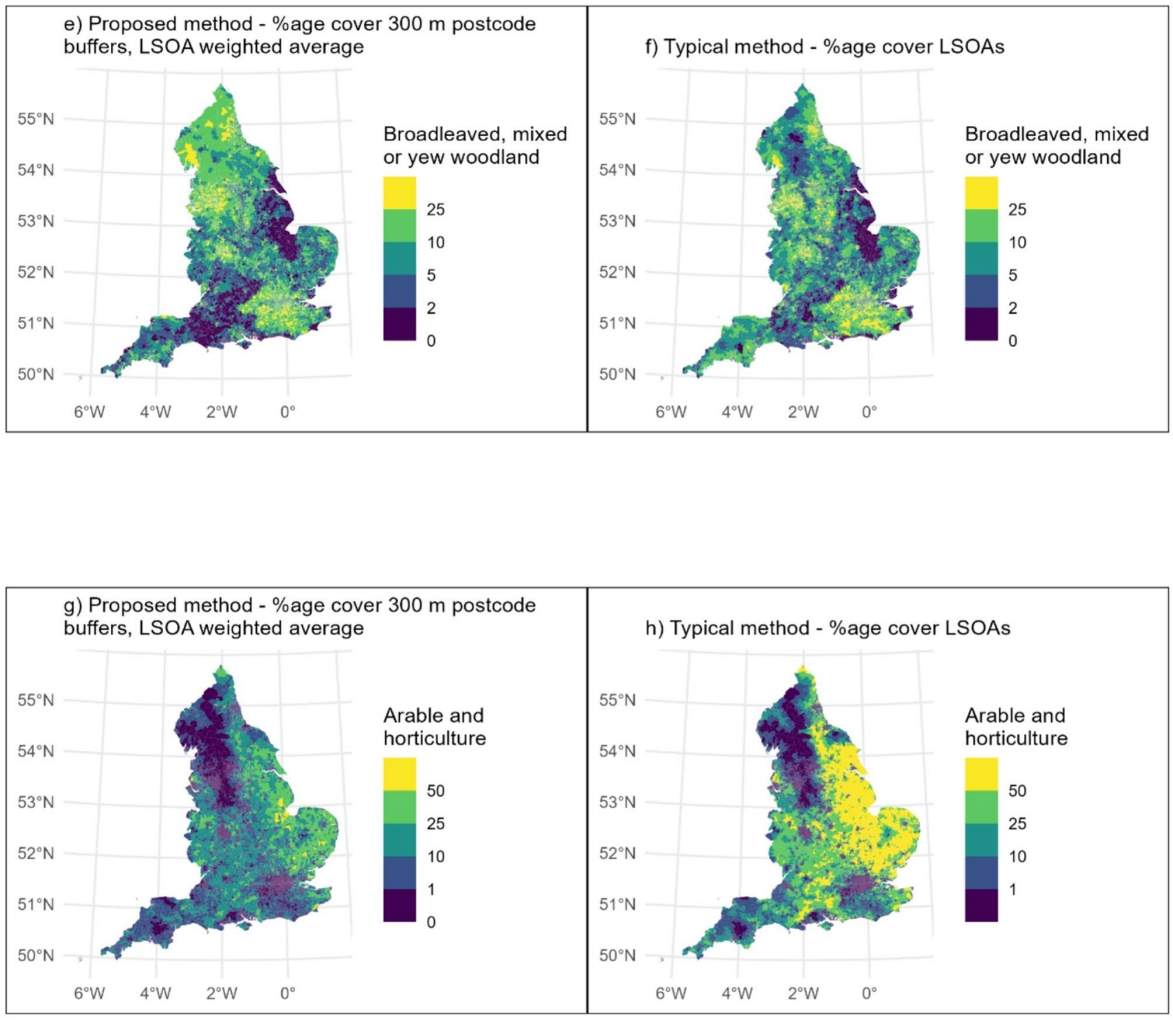

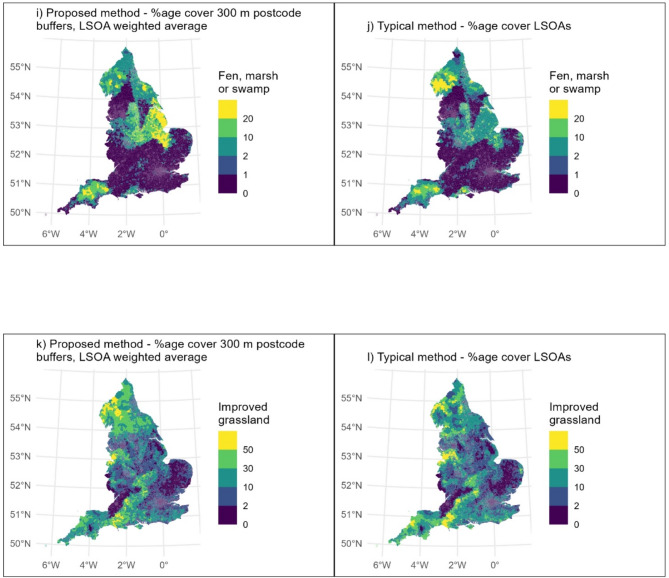
Comparisons of six land cover types as calculated using the proposed method (left hand side) and typical method (right hand side)

The most common land cover type was ‘built-up area and gardens’ for both our proposed method, and the typical method. The proportion tended to be higher when calculated using our proposed method, compared to the typical method (Table 2; Fig. 3). KS test results indicated significant differences in distributions for all land covers, with the exception of ‘coastal saltmarsh’ (Table 2).

However, there were 334 LSOAs where ‘built-up area and gardens’ made up > 99.9% of the total LSOA area using the typical method, whereas using our proposed method, only eight LSOAs were allocated ‘built-up area and gardens’ coverage of > 99.9%. This is to be expected, since LSOAs are intended to comprise as homogenous a neighbourhood as possible, delineated based on a range of factors, often utilising existing barriers such as roads (Supplemental Fig. 4). Therefore, for example, using the typical method, if an LSOA boundary runs along a major road next to a woodland, the LSOA across the road from the woodland would not be allocated any of that woodland as land cover, whereas using the proposed method postcode buffers would extend across the road and woodland land cover allocated to the LSOA.

**Fig. 4:**
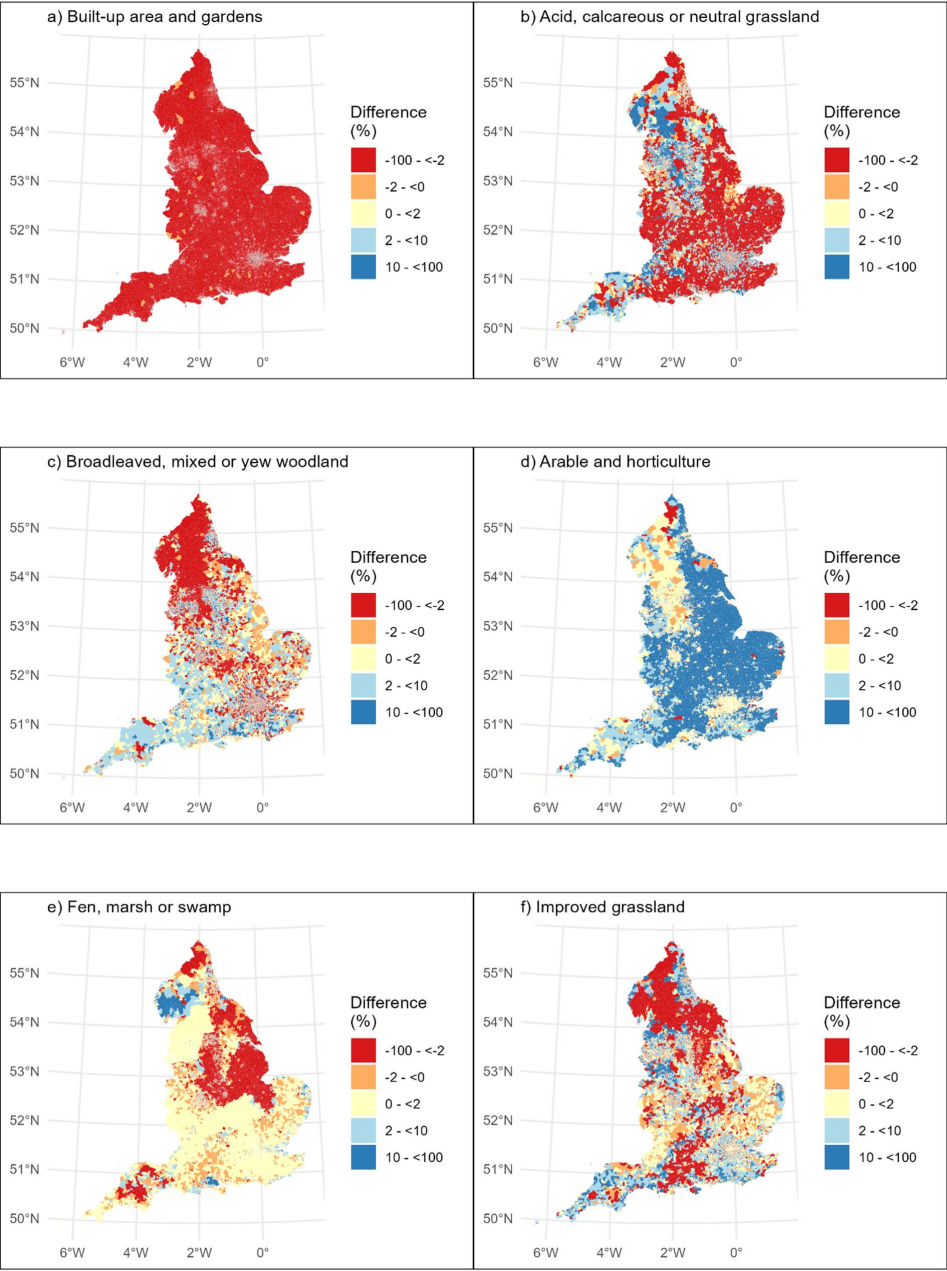
These show the difference in calculated percentage land covers (typical method – proposed method), such that where the value is positive, the typical method calculations are higher, when it’s negative, the proposed method calculations are higher

Using the proposed method, the five land cover types with the highest LSOA-averaged percentage coverage of postcode buffers were: ‘built-up area and gardens’; ‘acid, calcareous and neutral grassland’; ‘broadleaved, mixed and yew woodland’; ‘improved grassland’; and ‘fen, marsh or swamp’. This was predominantly the same when using the typical method. However, ‘arable and horticultural’ was in the top five whilst ‘fen, marsh or swamp’ was not. The percentage coverage for these six landcover types, and the difference between the two methods, are presented as maps (Figs. 3 and 4). In general, the percentage coverage of all non-built-up habitats was lower when calculated using the proposed method as compared to the typical method (Table 2). This indicates that, perhaps unsurprisingly, when considering the area near people’s homes (within 300 m), this tended to comprise more built-up area and less of all types of green and natural space than when considering LSOAs as a whole.

The maps demonstrate that this varies spatially. For example, in parts of northern England (Figs. 3 and 4), ‘broadleaved, mixed and yew woodland’ was at a higher percentage coverage when using the proposed method compared to the typical method. In contrast, in the Southwest, the percentage coverage of ‘broadleaved, mixed and yew woodland’ tended to be lower when using the proposed method, compared to the typical method (Fig. 4).

**Table 2:** Descriptive statistics of the habitat coverage as measured with our proposed method (LSOA weighted average of percent cover of 300 m postcode buffers) and the typical method (percentage cover of LSOAs)

Habitat	Proposed method					Typical method					KS test	
	Min	Max	Mean	SD	Median	Min	Max	Mean	SD	Median	Statistic	*p*
Built-up area and gardens	0.52	100.00	57.74	19.59	59.55	0.08	100.00	48.02	28.88	51.75	0.22	**< 0.001**
Acid, calcareous and neutral grassland	0.00	89.34	14.31	12.91	10.89	0.00	92.10	16.14	15.03	12.64	0.09	**< 0.001**
Broadleaved, mixed and yew woodland	0.00	76.54	12.71	10.28	10.75	0.00	91.01	13.63	12.09	10.80	0.05	**< 0.001**
Improved grassland	0.00	73.02	4.79	7.16	2.22	0.00	80.56	6.61	10.27	2.12	0.18	**< 0.001**
Fen, marsh or swamp	0.00	53.22	3.07	6.11	0.00	0.00	65.96	2.90	5.95	0.00	0.06	**< 0.001**
Arable and horticultural	0.00	61.94	2.86	6.49	0.00	0.00	90.08	7.24	15.84	0.00	0.12	**< 0.001**
Dwarf shrub heath	0.00	47.00	1.38	4.13	0.00	0.00	67.26	1.53	4.56	0.00	0.08	**< 0.001**
Scrub	0.00	45.85	1.21	3.84	0.00	0.00	52.59	1.11	3.81	0.00	0.04	**< 0.001**
Unclassified	0.00	61.31	0.53	1.83	0.00	0.00	58.63	0.65	2.33	0.00	0.08	**< 0.001**
Coastal sand dunes	0.00	42.71	0.38	1.90	0.00	0.00	64.84	0.55	3.03	0.00	0.02	**< 0.001**
Water	0.00	29.43	0.21	0.97	0.00	0.00	79.57	0.46	2.20	0.00	0.05	**< 0.001**
Estuaries	0.00	44.01	0.19	1.67	0.00	0.00	1.47	0.00	0.01	0.00	0.03	**< 0.001**
Bare sand	0.00	25.55	0.18	0.79	0.00	0.00	53.72	0.34	1.67	0.00	0.06	**< 0.001**
Sea	0.00	31.10	0.17	1.27	0.00	0.00	35.04	0.03	0.37	0.00	0.02	**< 0.001**
Coniferous wood	0.00	25.50	0.14	0.83	0.00	0.00	62.98	0.41	2.27	0.00	0.05	**< 0.001**
Bracken	0.00	19.99	0.07	0.42	0.00	0.00	36.77	0.14	0.86	0.00	0.02	**< 0.001**
Bog	0.00	17.12	0.04	0.33	0.00	0.00	56.38	0.15	1.54	0.00	0.02	**< 0.001**
Coastal saltmarsh	0.00	14.81	0.01	0.21	0.00	0.00	64.55	0.07	1.14	0.00	0.01	0.190
Bare ground	0.00	7.64	0.01	0.10	0.00	0.00	19.34	0.03	0.30	0.00	0.02	**< 0.001**

### Correlation between habitat cover by the two methods

Overall, Kendall’s tau ranged from 0.56 (Water) to 0.89 (Scrub; Table 3). Correlation varied when exploring urban and rural LSOAs separately. For example, when looking at ‘broadleaved, mixed and yew woodland’, the two methods were less similar in rural areas compared to urban areas (urban Kendall’s tau = 0.73 *p* < 0.001; rural Kendall’s tau = 0.59, *p* < 0.001). This was not the case for all habitats. For example, for ‘built-up area and gardens’, in urban areas Kendall’s tau = 0.65 (*p* < 0.001) and in rural areas Kendall’s tau was similar at 0.67 (*p* < 0.001; Table 3).

**Fig. 5:**
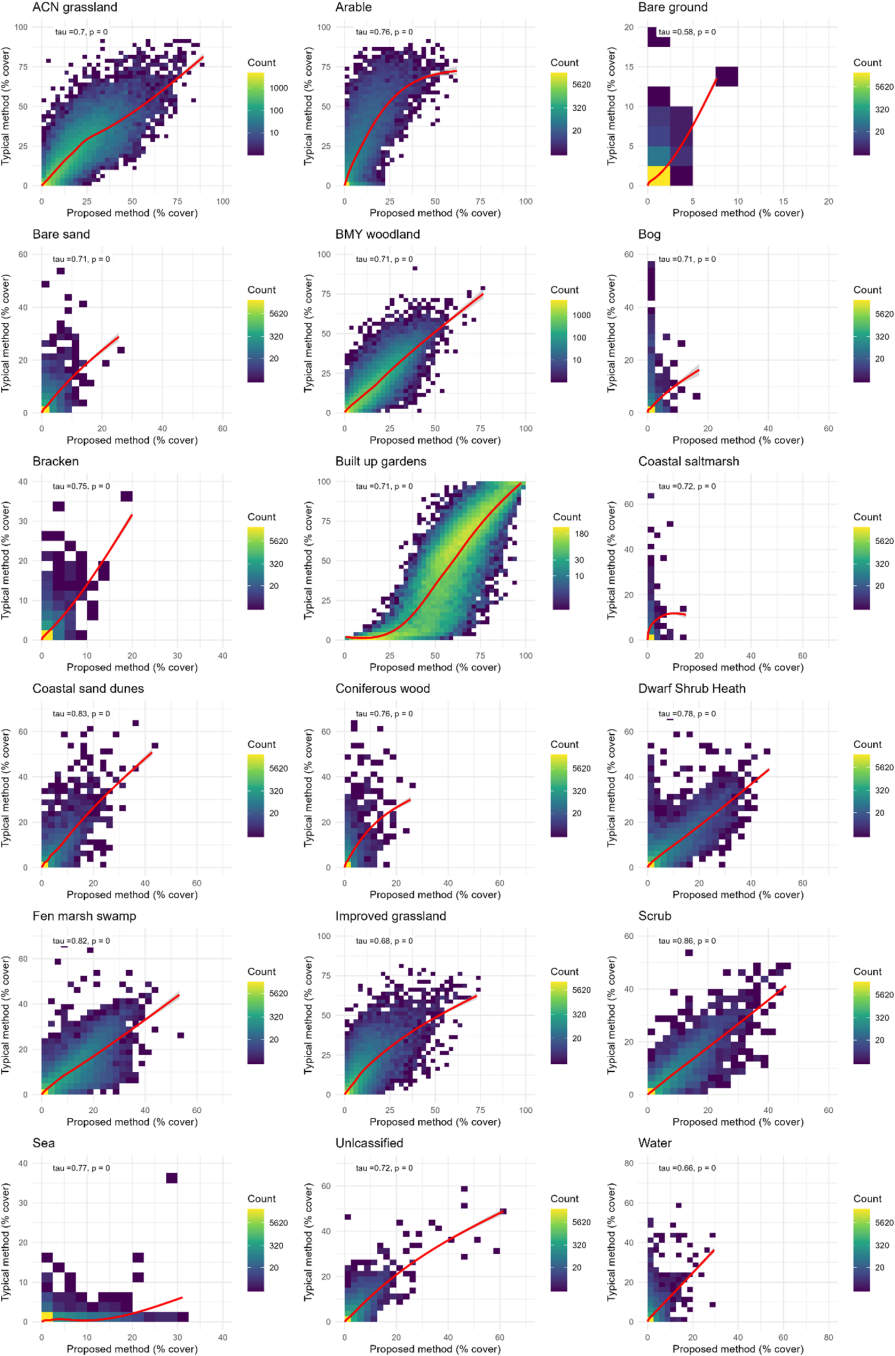
Correlation for each habitat coverage when calculated using the two different methods. The line of best fit presented is generated with a generalised additive model (GAM) smoothing function (geom_smooth(method = “gam”)). Estuaries are excluded due to very low numbers

Figure 5visualises the correlation. ‘Scrub’ has the highest correlation, and this is visualised with a linear line of best fit, whereas ‘arable and horticulture’ has a non-linear correlation. The typical method tends to estimate higher coverages of arable land cover than our proposed method, but the difference tends to decrease with increasing cover. Conversely, percentage cover of ‘built-up area and gardens’ tends to be lower when calculated with the typical method compared to our proposed method. However, the difference decreases with increasing cover (Fig. 5).

**Table 3:** Kendal’s Tau correlation between the habitat coverage as calculated our proposed method (percentage cover within 300 m postcode buffers, averaged at the LSOA level and weighted by the number of domestic delivery address) and the typical method (percentage land cover of the LSOA)

Habitat	All LSOAs		Rural LSOAs		Urban LSOAs	
	tau	*p*	tau	*p*	tau	*p*
Scrub	0.89	< 0.001	0.89	< 0.001	0.85	< 0.001
Coastal sand dunes	0.88	< 0.001	0.88	< 0.001	0.82	< 0.001
Sea	0.81	< 0.001	0.81	< 0.001	0.74	< 0.001
Bare sand	0.80	< 0.001	0.80	< 0.001	0.67	< 0.001
Dwarf shrub heath	0.80	< 0.001	0.80	< 0.001	0.77	< 0.001
Fen marsh swamp	0.78	< 0.001	0.78	< 0.001	0.83	< 0.001
Bog	0.77	< 0.001	0.77	< 0.001	0.69	< 0.001
Coastal saltmarsh	0.77	< 0.001	0.77	< 0.001	0.66	< 0.001
Estuaries	0.76	< 0.001	0.76	< 0.001	0.66	< 0.001
Bracken	0.75	< 0.001	0.75	< 0.001	0.69	< 0.001
Unclassified	0.73	< 0.001	0.73	< 0.001	0.72	< 0.001
Improved grassland	0.68	< 0.001	0.68	< 0.001	0.65	< 0.001
Arable	0.67	< 0.001	0.67	< 0.001	0.69	< 0.001
Built-up area and gardens	0.67	< 0.001	0.67	< 0.001	0.65	< 0.001
Coniferous wood	0.67	< 0.001	0.67	< 0.001	0.71	< 0.001
Acid, calcareous or neutral grassland	0.60	< 0.001	0.60	< 0.001	0.71	< 0.001
Broadleaved, mixed or yew woodland	0.59	< 0.001	0.59	< 0.001	0.73	< 0.001
Bare ground	0.58	< 0.001	0.58	< 0.001	0.59	< 0.001
Water	0.56	< 0.001	0.56	< 0.001	0.65	< 0.001

### Difference by size class

**Fig. 6:**
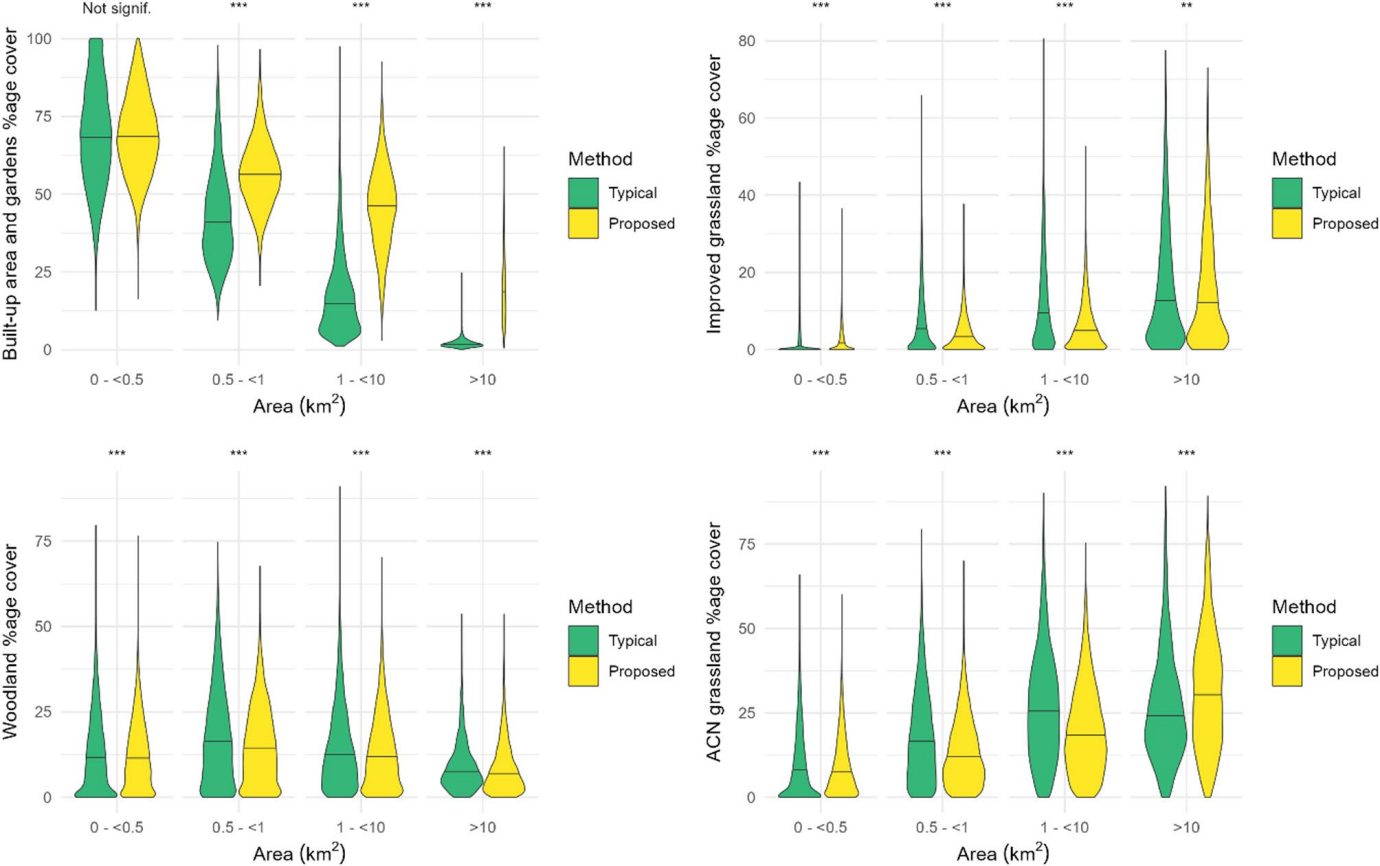
Distribution of percentage land cover as calculated by the two methods. Where the typical method is the percentage cover of each land cover within LSOAs and the proposed method is the LSOA-averaged percentage cover of 300 m postcode buffers, weighted by the number of domestic delivery addresses. The stars indicate the significance levels of the Mann-Whitney U test for differences in medians, where: ***=*p* < 0.001, **=*p* < 0.01, *=*p* < 0.05. “Woodland” represents the Broadleaved, mixed and yew woodland habitat and “ACN grassland” represents the Acid, calcareous and neutral grassland habitat

The differences between the estimated land covers by the two methods vary with size of LSOA (Fig. 6). We present size classes on a logarithmic scale, but split the smallest size class as most LSOAs were in this size class. In all size classes ≥ 0.5km^2^, the percentage cover of ‘built-up area and gardens’ is greater using our proposed method than the typical method. For the smallest size class presented, < 0.5km^2^, there was no significant difference in the medians as calculated by the two methods. For ‘improved grassland’, statistical tests indicated differences for all size classes, although the nature of the difference varied with size class. For the smallest LSOAs, the proposed method resulted in a higher calculation for the land cover, whereas, for the middle two size classes, the typical method resulted in a higher calculation for ‘improved grassland’. This pattern was also observed in ‘acid, calcareous, and neutral grasslands’. Within the smallest size class, there was little difference in the land cover calculations between the two methods. However, in the middle two size classes, the typical method produced higher land cover estimates. Conversely, in the largest size class, our proposed method resulted in higher land cover estimates. There were small but significant differences in coverage estimates of ‘broadleaved, mixed and yew woodland’ for all size classes (Fig. 6).

### Difference by region

**Fig. 7:**
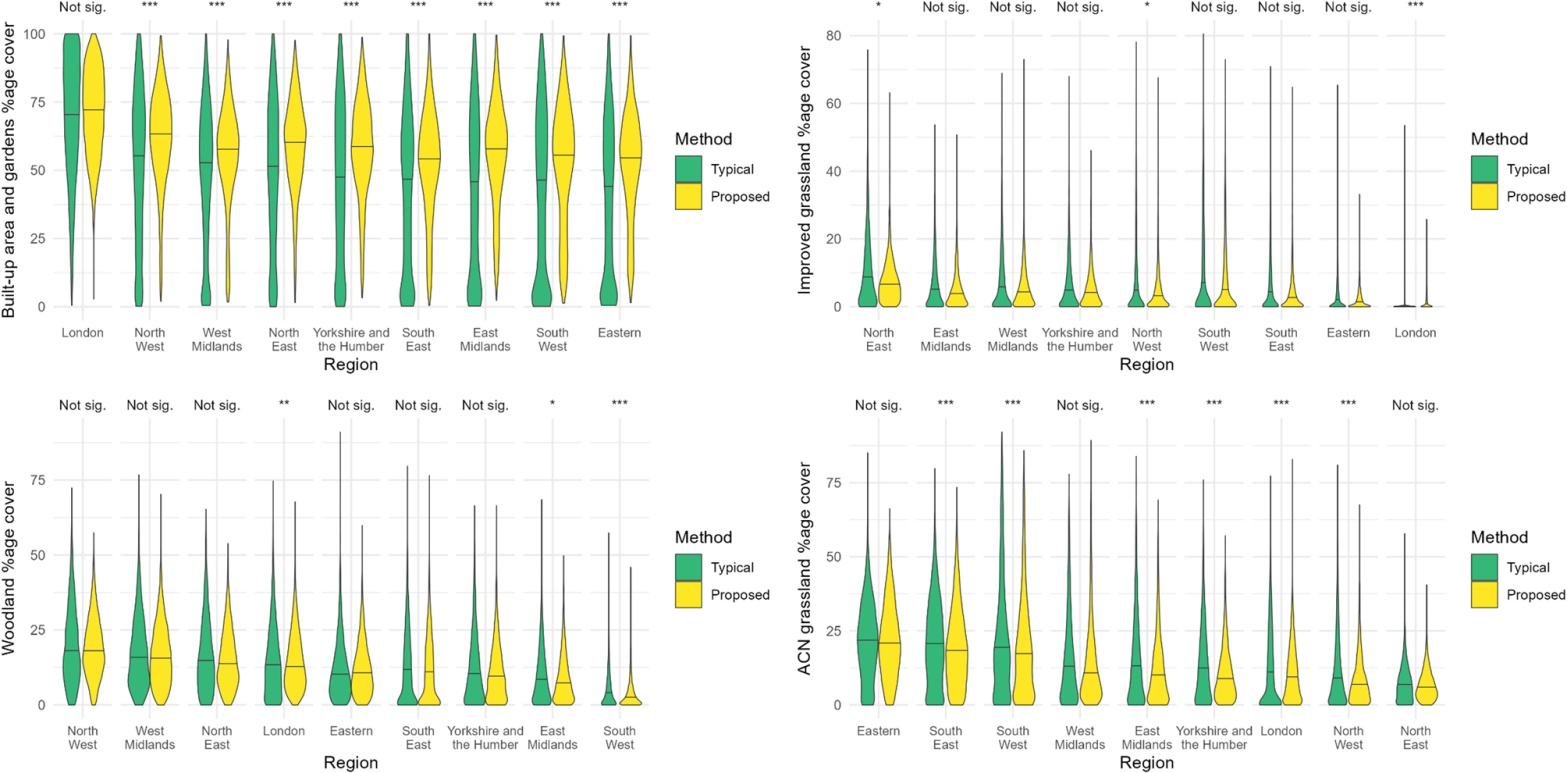
Distribution of percentage land cover as calculated by the two methods by region of England. Where the typical method is the percentage cover of each land cover within LSOAs and the proposed method is the LSOA-averaged percentage cover of 300 m postcode buffers, weighted by the number of domestic delivery addresses. The stars indicate the significance levels of the Mann-Whitney U test for differences in medians, where: ***=*p* < 0.001, **=*p* < 0.01, *=*p* < 0.05. “Woodland” represents the Broadleaved, mixed and yew woodland habitat and “ACN grassland” represents the Acid, calcareous and neutral grassland habitat

The differences between the estimated land covers by the two methods also vary with region of the country (Fig. 7). For estimating built-up area and gardens, the typical method was significantly lower for all regions, with the exception of London where the difference was not significant (it is notable that London is the only region whose land area is almost entirely ‘urban’). In general, the non-built-up land covers were lower as calculated using the proposed method in comparison to the typical method, but significant differences in the distributions varied by region (Fig. 7).

## Discussion

In this study, we propose a method for characterising the environment at the small area level that aims to better represent the environmental exposures individuals encounter around their homes, maintaining consistency across varying geographical unit sizes. This method is adaptable and could be applied in other country contexts where population counts are available for smaller geographical units within the main units of analysis. Within the UK context, it could also be applied to other land cover datasets beyond the Living England Habitat Map utilised here, provided researchers have access to the boundaries of the required geographical areas and the Ordnance Survey Code-Point dataset or other comparable postal information datasets. The approach can also be applied to any spatial assessment of environmental exposure with sufficiently high resolution at the LSOA level so as to be useful in reducing exposure misclassification e.g. for air pollution.

Our results indicate that overall, using our proposed method, individuals tend to be allocated higher exposure to built-up areas and gardens compared to the typical method, while their exposure to natural and vegetated land covers is reduced when using our proposed method. However, we find that the variation depended on the region of the country, urban/rural status, land cover type and LSOA size class.

Other attempts have been made to improve the exposure assessment at the small statistical area level. For example, a buffer around the population weighted centroid has been used, which provides a point location per LSOA based on the population density within the LSOA [37]. Our method improves on this approach by utilising all the postcode locations within an LSOA, rather than a single point. Another approach has been to use a gridded population dataset at a resolution of 100 m to weight land covers within an administrative boundary [61]. This approach could be useful if postal locations (which can be more precise) are not available. Other similar approaches can also be found. For example, in Mears et al. [9],, they used a dataset of residential addresses and averaged the number of trees within a 100 m buffer of each within an LSOA. However, our proposed method is designed to be used where the residential addresses are not known, or where processing of this at a national scale would be prohibitively time consuming, and we weight the percentage land cover within postcode buffers by the number of domestic delivery addresses. Perhaps the most comparable approach is described by Brindley et al. [53], who averaged modelled air pollution levels from a spatial grid at postcode point locations in Sheffield. They aggregated data at the enumeration district (ED) level, used as the smallest units for reporting UK census data between 1961 and 1991 [62]. They calculated a buffer region by taking 1 km buffer around each postcode point within the ED, then calculated point averages from all postcode points within this buffer region, weighted by the number of domestic delivery addresses at each point. In our study, we calculated land cover percentages within each individual postcode buffer, and then averaged these values by LSOA, weighted by the number of domestic delivery addresses. While the methods and aims are somewhat different, our approach is conceptually similar.

### Implications

This work has implications for future research assessing the relationship between exposure to green and blue spaces at the small area level with health and well-being outcomes. The work adds to previous studies which identify differences in the geographical metrics used to estimate exposure as responsible for variations in associations between greenspace and health [63].

We found differences in the estimations by size class and land cover. Assuming that our approach better reflects the environment that people experience on a daily basis, our results suggest that the typical method underestimates the built-up areas that people are exposed to, while differentially estimating non-built-up land covers depending on size class. Specifically, the typical method tends to overestimate non-built-up land covers overall, whilst underestimating them in the largest LSOAs. This may help to explain some of the mixed results found in the literature [64–66].

### Strengths and limitations

Our proposed method offers several advantages. It mitigates edge effects by allowing environmental exposure to extend beyond the LSOA boundary through the use of a 300-metre postcode buffer and maintains consistency across varying LSOA sizes, which mitigates the issue of a variably sized administrative area. Together, these improvements better represent the environmental exposure people experience in their daily lives. Further research will assess the association with health and well-being outcomes.

However, limitations in the method remain. The method relies on point postcode locations, as provided in the Ordnance Survey Code-Point dataset and are based on the mean location of individual dwellings within that postcode. The number of dwellings with the same postcode can vary, as can the area that the postcode includes. Therefore, there remains the possibility of exposure misclassification for some individuals and populations [33]. However, other researchers have found that offsets of postcode locations by few hundred metres or less are unlikely to result in different conclusions as for the original locations [30]. Further, our method averages across all postcode buffers within an LSOA. There is therefore the possibility of exposure misclassification depending on the individual postcode of residence within an LSOA. However, this is reduced by weighting the averaging by the number of domestic delivery addresses. Within rural areas, where postcode areas are further apart, this risk is increased. However, by utilising postcode locations we are reducing this risk in comparison to the average across the whole LSOA. In more rural areas we avoid allocating exposures to land covers where people are unlikely to live, unlike where quantity is assessed using the typical method averaging across the whole LSOA, regardless of where people live. The modifiable areal unit problem also affects such spatial statistics, and the proposed method does not eliminate this issue (this describes the bias that results from the selection of the areal unit at which data is aggregated) [30].

An alternative approach would be to utilise building level data where this is available, calculate the habitat coverage around individual dwellings and averaging this at the LSOA level. This could be suited for researchers working on a smaller study area, because this would require considerably more data processing time. Another alternative would be to estimate land cover within postcode polygons, instead of postcode point buffers. We chose not to do this because this would still result in analytical areas of varying size due to dwelling density in a similar way as averaging at LSOA level. Nevertheless, it may be a suitable approach if a study area consists of dwellings at similar densities.

Exposure misclassification may also occur due to the underlying data. In this example we use the Living England dataset, of which the spatial resolution is 10 m and the modelling accuracy is provided as 88% although this varies with habitat type [49]. Here, we applied a buffer distance of 300 m, this is consistent with other studies [3, 27, 67, 68] and policy indicators [56, 57]. However, depending on the outcome being assessed future research could explore other appropriate buffer sizes or incorporate a distance decay function [38, 63].

## Conclusion

We introduce a novel methodology to more realistically calculate exposure to different types of land cover at the small statistical geography level. We illustrate the proposed method by comparing it with the more typical percentage coverage of LSOAs, using the Living England habitat map. Overall, exposure to non-built-up areas was generally lower using our proposed method compared to averaging across LSOAs as a whole, while the opposite was true for built-up areas. However, we find differences by region of the country, urban/rural status, land cover type and LSOA size class. This adds further evidence indicating the uncertainty in mental health and wellbeing outcomes due to misclassification.

Our method could be applied to other contexts or data where the spatial environmental maps, the postal locations and the small statistical geography boundaries exist or are accessible. We suggest that it represents an improved method and may reduce environmental exposure misclassification associated with variable unit size when conducting research at the small statistical geography level.

## Supplementary Information

Supplementary Material 1

## Data Availability

The datasets supporting the conclusions of this article are available in the Zenodo repository, at [https://zenodo.org/records/14998734](https:/zenodo.org/records/14998734)The code is available at [https://github.com/j-k-garrett/RENEW\_mapping](https:/github.com/j-k-garrett/RENEW_mapping).
