# Supplementary material for: A novel approach for mapping exposure to land cover at the small statistical geography level
Source: Int J Health Geogr. 2025 Nov 25;24:37. doi: 10.1186/s12942-025-00425-7 (PMC12648878; doi:10.1186/s12942-025-00425-7)
Supplement: Supplementary file 1 — Supplementary Material 1 [file 12942_2025_425_MOESM1_ESM.docx]

**Supplemental Materials**


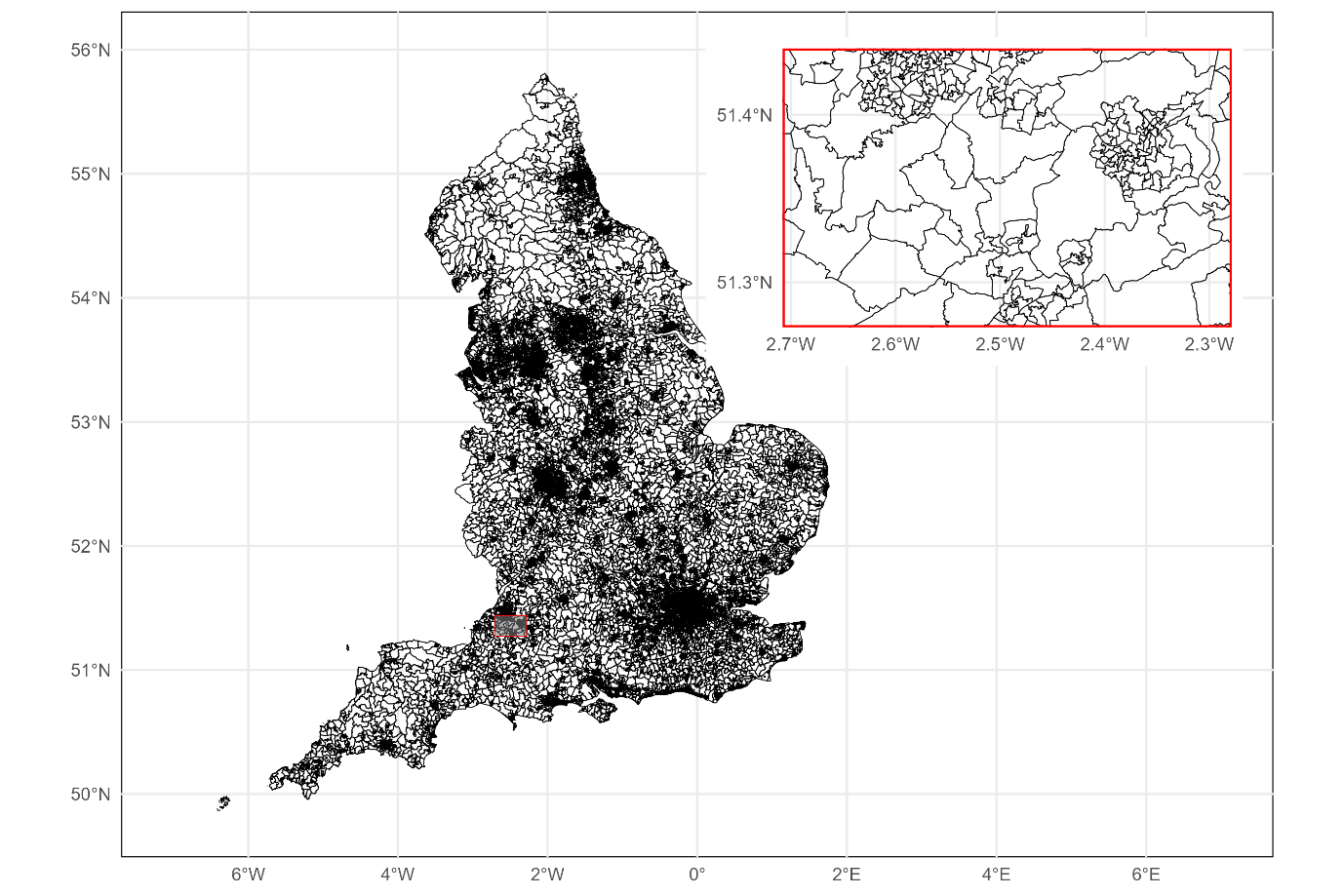


Figure 1 LSOAs with inset map showing Bath area and the differences in sizes between LSOAs


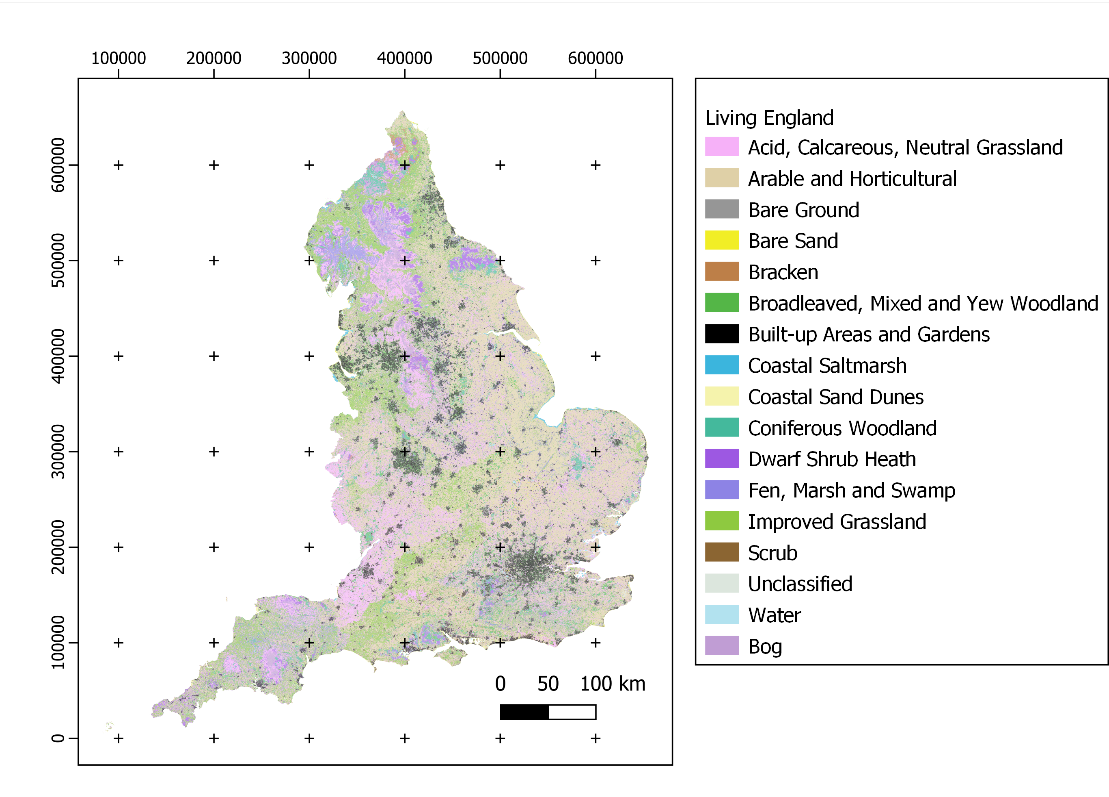


Figure 2 Living England Habitat map (Natural England 2022)


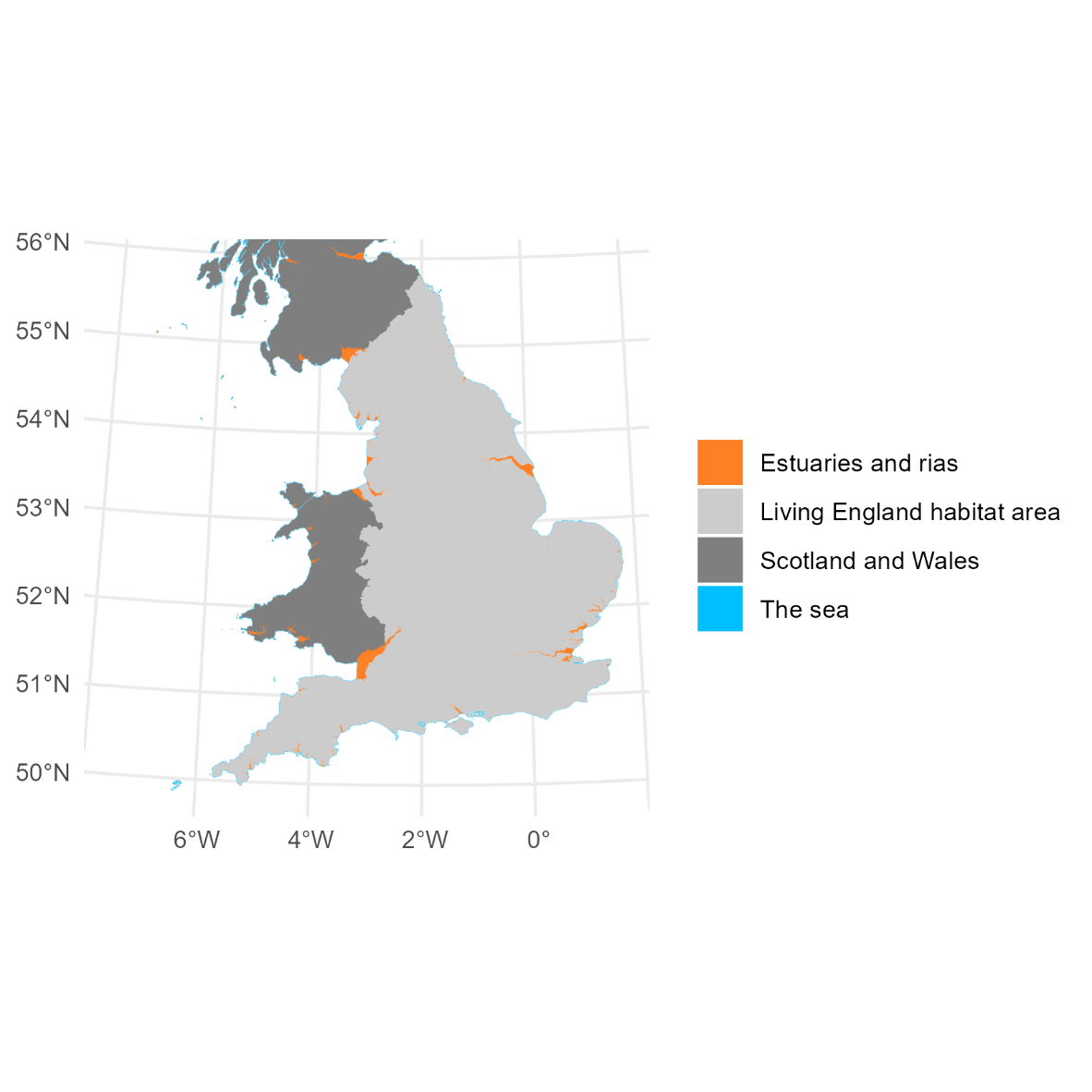


Figure 3 Additional data including estuaries and rias (Joint Nature Conservation Committee 2018a; Joint Nature Conservation Committee 2018b); Wales and Scotland boundaries (Ordnance survey 2022)

*
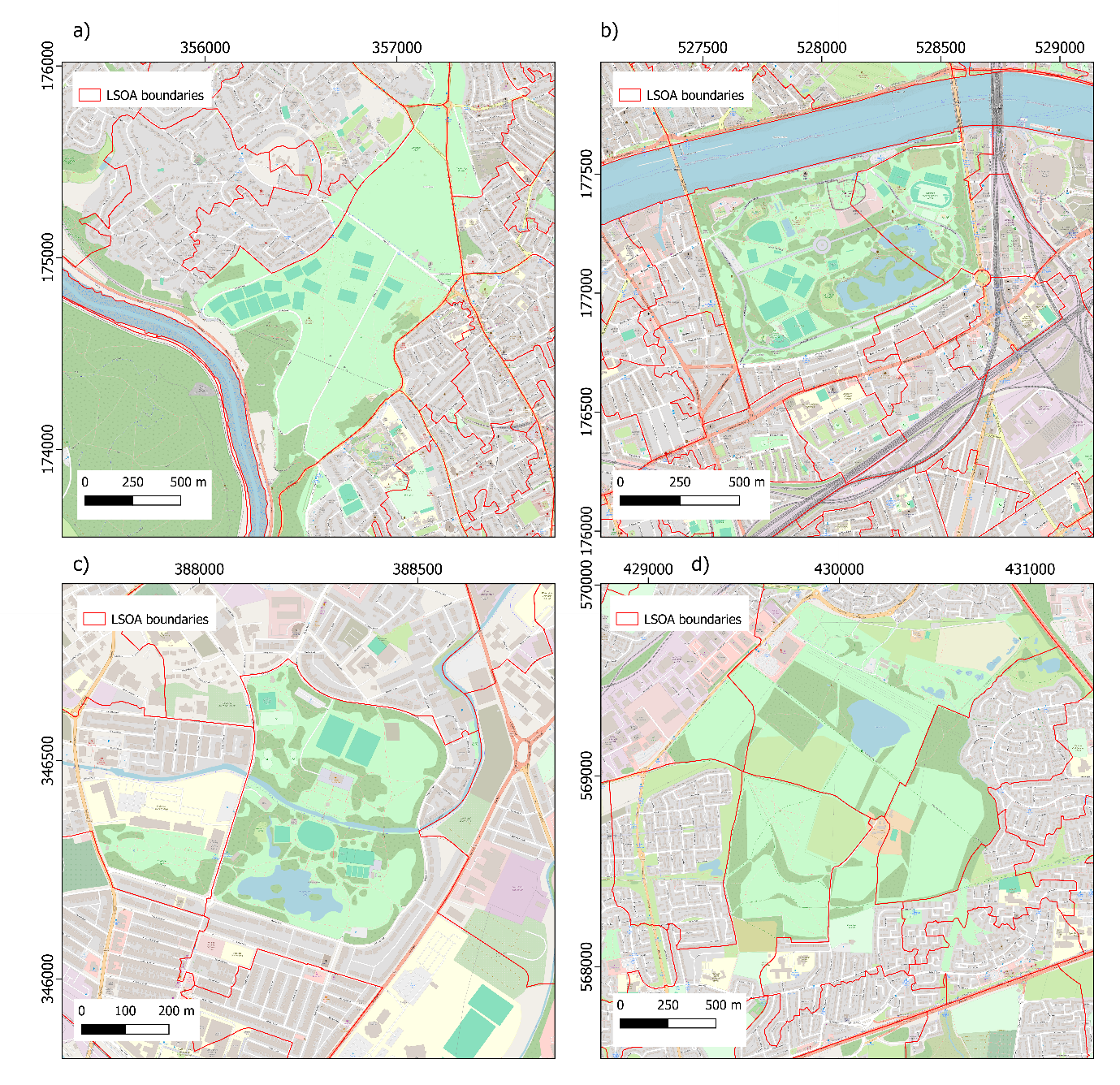
*

Figure 4 Example LSOA boundaries in a) Bristol, surrounding Clifton Downs; b) London, surrounding Battersea Park; c) Stoke-On-Trent surrounding Hanley Park; and d) Newcastle Upon Tyne, surrounding the Rising Sun Country Park
